# Serum neopterin levels in patients with replicative and nonreplicative HBV carriers

**DOI:** 10.1186/1471-2334-6-157

**Published:** 2006-10-31

**Authors:** Ilknur Kaleli, Melek Demir, Nural Cevahir, Mustafa Yılmaz, Suleyman Demir

**Affiliations:** 1 Department of Microbiology, Pamukkale University Medical Faculty, Denizli, Turkey; 2 Department of Gastroenterology, Pamukkale University Medical Faculty, Denizli, Turkey; 3 Department of Biochemistry, Pamukkale University Medical Faculty, Denizli, Turkey

## Abstract

**Background:**

Infection by hepatitis B virus (HBV) causes complicated biochemical, immunological and histological changes in host immune response against the virus which can be specific or non-specific. Recent attention has focused on neopterin as a marker for the activation of cell mediated immunity. The aim of this study was to define the pattern of neopterin levels in replicative and nonreplicative HBV carriers.

**Methods:**

Thirty HBV replicative carriers and 25 nonreplicative HBV carriers and 30 healthy adult patients were included this study. Hepatitis markers were determined by commercial kit based on chemilumminesans assay. HBV DNA was quantified by hybrid capture system. Serum neopterin levels were measured by the method of competitive enzyme-linked immunosorbent assay. Results were expressed as mean ± SD and ranges.

**Results:**

In the nonreplicative group, except for one patient, all the patients' HBeAg were negative and anti-HBe were positive. That particular patient was HBeAg positive and anti-HBe negative. In the replicative group, 23 out of 30 patients have positive HBeAg and negative anti-HBe; 7 out of 30 patients have negative HBeAg and positive anti-HBe. Serum neopterin concentrations were 14.5 ± 10.0 (4.2–41) nmol/L in replicative HBV carriers, 8.9 ± 4.3 (2.1–22) nmol/L in nonreplicative HBV carriers and 7.1 ± 2.2 (4.0–12) nmol/L in the control group. Serum neopterin levels and the rates of abnormal serum neopterin levels in the replicative group were higher than the control group (*P < 0.01 and P < 0.05*). In the nonreplicative group, serum neopterin levels were not different from those of the control. There was a difference between replicative and nonreplicative groups in the respect of neopterin levels.

**Conclusion:**

In the hepatitis B infected carriers, elevated neopterin levels may be an indicator of the presence of replication.

## Background

Hepatitis B virus (HBV) infection is a worldwide health problem. The clinical consequences of hepatitis B virus infection are extremely variable, including clinical syndromes such as fulminant, acute and chronic hepatitis, hepatocellular carcinoma, and the asymptomatic carrier state [1]. Infection by hepatitis B virus causes complicated biochemical, immunological and histological changes in host immune response against virus can be specific or nonspecific. Nonspecific response occurs via cytokines or other substance.

Neopterin is a pyrazino-pyrimidine derivative, namely 2-amino 4-hydroxy-(1',2',3' trihydroxypropyl) pteridine. Biosynthetically neopterin derives from guanosine triphosphate [2]. Neopterin is produced by activated macrophages, in response to interferon-gamma derived from activated T cell. Recent attention has focused on neopterin as a marker for the activation of cell mediated immunity [3]. Elevated neopterin levels in serum and urine have been reported in several acute and chronic infections, allograft rejection, autoimmune and malignant diseases [2,4-7].

In some viral infections neopterin concentration increases during the incubation period, shows a pronounced peak during clinical symptoms and sharply decreases and normalizes during the period of convalences, when neutralizing antibody titers become measurable in the circulation [2].

The aim of this study is to define the pattern of neopterin levels in replicative and nonreplicative HBV carriers.

## Methods

### Patients

This study included 55 patients infected with HBV for more than 6 months and 30 healthy adult subjects as a control group. All patients gave verbally informed consent to participate in the study. A total of 30 HBV DNA (+) patients were between the age of 13 and 73 years in the replicative group, 25 HBV DNA (-) patients between 20 and 71 were included in the nonreplicative group. The average age of 13 female and 17 male individuals in the replicative group were 40.8 ± 15.3 (13–73). In the nonreplicative group, the average age of 10 female and 15 male individuals was 42.8 ± 13.4 (20–71). There are 30 health subjects in the control group consisted of 12 females and 18 male subjects. The average age of controls was 44.1 ± 13.6 (21–68) years. The groups showed no significant differences in age and sex. The diagnostic criterias for HBV infection were sero-positivity for hepatitis B surface antigen (HBsAg), lack of anti-hepatitis B surface antibodies (anti HBs) and presence of anticore IgG antibodies (anti-HBc). If serum HBV DNA levels was higher than 5 pg/ml by hybrid capture system, the patients were considered as HBV replicative carriers [8,9]. Of the 55 patients, 30 were HBV replicative carriers, 25 were nonreplicative carriers when serum HBV-DNA undetectable by hybrid capture system.

The control group included 30 healthy individuals without HBV infection (HBsAg negative, antiHBs negative and anti HBc IgG negative). All patient and controls were without hepatitis C, hepatitis D, Human immunodeficiency virus (HIV) infections and systemic bacterial or fungal infection and autoimmune diseases. Other causes of liver disease such as alcohol consumption and autoimmune hepatitis were excluded.

### Procedures

Viral markers (Access Beckman Coulter-Biorad, USA) and biochemical parameters Synchron LX20, Beckman Coulter, Brea, CA, USA) were determined by commercial kits. HBV DNA was quantified by hybrid capture system (Digene Diagnostic MD, USA), according to the manufacturer's instruction.

### Serum neopterin levels

Serum neopterin levels were measured following the basic principle of competitive enzyme-linked immunosorbent assay (Neopterin ELISA, Immuno Biological Laboratories, Hamburg, Germany): the competition between a peroxidase-conjugated and nonconjugated antigen for a fixed number of antibody binding sites (rabbit anti-neopterin). The peroxidase-conjugated antigen-antibody complexes bind to the wells of the microtiter strips, which are coated with a goat anti-rabbit antibody. Unbound antigen was removed by washing. After the substrate reaction the optical density was measured at 450 nm. Quantification of the samples were estimated by comparing the enzymatic activity of the samples with a response curve prepared by using standards ranging 0 to 111 nmol/L. Results were expressed as mean ± SD and ranges. Serum specimens were stored at -20°C in the dark until the assay time.

### Statistical analysis

For statistical analysis, Kruskal Wallis and Bonferroni post-hoc tests were used. A p value of <0.05 was considered significant.

## Results

**Figure 1:**
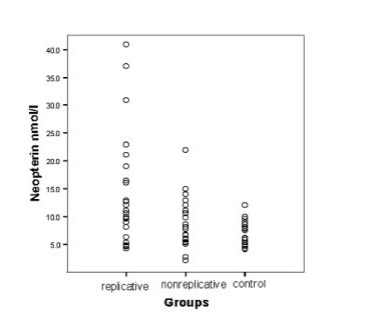
Serum neopterin levels in patients with replicative, nonreplicative and control.

The means of serum neopterin concentrations were 14.5 ± 10.0 (median: 11.5 ranged 4.2–41) nmol/L in replicative HBV carriers, 8.9 ± 4.3 (median: 8.0 ranged 2.1–22) nmol/L in nonreplicative HBV carriers and 7.1 ± 2.2 (median: 7.5 ranged 4.0–12) nmol/L in the control group. The difference between the replicative, nonreplicative and control was statistically significant (* P < 0.05*). In the replicative carriers neopterin levels (Table 1) and rates (Table 2) were significantly higher than those of control (*P < 0.01 and P < 0.05*). Also, serum neopterin levels in replicative group were higher than in nonreplicative groups (*P < 0.05*). There are no difference between men and women. The levels of serum neopterin in 30 control and 30 replicative and 25 nonreplicative patients were shown in Table 1 and 2 and Figure 1. In this study, when the cut-off value was set as 8.7 nmol/L, 21 out of 30 replicative patients, 11 out of 25 nonreplicative patients and 11 out of 30 controls' neopterin values were higher than this level (p < 0,05). On the other hand, when the cut-off value was set as 10 nmol/L, 18 out of 30 replicative, 8 out of 25 nonreplicative and 5 out of 30 control subjects' neopterin values were found to be higher than this point (p < 0,05) (Table 2).

**Table 1:** Serum neopterin levels in replicative, non replicative carriers and control

Groups	Neopterin Levels (nmol/L)				
	
	Mean	Median	Standard Deviation	Minimum	Maximum
Replicative (n = 30)	14.5^a, b^	11.5	10.0	4.2	41
Non-replicative (n = 25)	8.8	8.0	4.3	2.1	22
Controls (n = 30)	7.1	7.5	2.2	4.0	12

a:*P < 0.01 *vs controls, b: *P < 0.05 *vs nonreplicative

**Table 2:** Rates of Abnormal Serum Neopterin Levels in Various Groups

Groups		Abnormal Serum Levels			
		
	No of cases	≥ 8,7 nmol/L		≥ 10 nmol/L	
		n	%	n	%
Replicative	30	21^a^	70	18^a^	60
Non-replicative	25	11	44	8	32
Controls	30	11	36,6	5	16,6

a: *P < 0.05 *vs controls

Neopterin levels in nonreplicative carriers did not differ from those of control. When HBV-DNA levels were categorized according to copy values as picogram, neopterin levels were not correlated HBV-DNA levels (Table 4).

**Table 4:** Serum neopterin levels according to HBV DNA in replicative group

HBVDNA (pg)	Mean	n	Std. Deviation	Median	Minimum	Maximum
5–100	17.7	8	10.2	14.0	9.5	41.0
101–500	12.7	7	9.1	10.5	4.0	31.0
501–1000	17.8	2	18.7	17.8	4.6	31.0
1000<	12.3	13	10.1	8.0	4.2	37.0

In the nonreplicative group, except for one patient, all the patients' HBeAg were negative and anti-HBe were positive. That particular patient's HBeAg was positive and anti-HBe negative. In the replicative group, 23 out of 30 patients have positive HBeAg and negative anti-HBe; 7 out of 30 patients have negative HBeAg and positive anti-HBe.

The averages of ALT levels were in reference limits according to our laboratory for all groups. The mean of serum ALT levels were 44 ± 14, 29 ± 11 and 26 ± 7.5 IU/L in the replicative, nonreplicative carriers and control group, respectively. Serum ALT levels were not different according to interquartile range of serum neopterin (Table 3).

**Table 3:** Serum ALT levels according to interquartil range of serum neopterin

Neoptein Quartile	n	Mean	Std. Deviation	Median	Minimum	Maximum
1	21	35.85	14.31	29	13	61
2	22	30.04	11.79	28.5	14	62
3	25	31.52	13.77	25.0	18	63
4	17	38.17	14.54	35.0	14	65

p > 0.05

## Discussions and conclusion

Immune defense against virus infection involves both nonspecific and antigen-specific phases [1]. The stimulation of the cellular immunity associated with macrophage activation causes an increase of neopterin in the urine, serum and other body fluids. Neopterin has been reported as the indicator of local macrophage activity in different body fluid. Neopterin secretion is found to increase in patients suffering from viral, bacterial or parasitic infections and with immune and autoimmune diseases [2,3,10]. Neopterin is considered as a parameter of immun activation and inflammation [11]. Elevation of neopterin levels in AIDS and tumor patients correlates with the severity of the disease [12-14].

The measurement of HBV DNA levels in serum has become an important tool for the identification of individuals with high levels of viral replication that might benefit from antiviral therapy, monitoring of patients on therapy, and prediction of whether antiviral therapy will be successful. Several molecular approaches, commercially available tests, have been used to quantity serum HBV DNA levels [15]. Hybrid capture method was used in this study to assess the viral load of the patient group. In this study, if patients have >5 pg/ml viral load they are called replicative; if the patients have not HBV DNA they are called nonreplicative. In the present study serum neopterin levels were examined in replicative HBV carriers and nonreplicative HBV carriers. In the replicative HBV carriers, the neopterin levels were found to be elevated. Serum neopterin levels in the replicative group were significantly higher than those of control group (*P < 0.01*).

Neopterin concentrations in serum show a slight increase with age but not sex dependent difference [2,10,16]. As the upper limit of serum neopterin in healthy controls which are age between 19–75 years, 8.7 nmol/L are accepted in some studies[2,10]. The cut-off value of our kit is 10 nmol/L. We estimated of 83,3^th ^percentile of neopterin values of controls as <10 nmol/L. In present study, 63,3^th ^percentile of neopterin values of controls were estimated as <8.7 nmol/L Therefore we decided that our cut-off value as 10 nmol/L.

It is reported that clinically healthy HBsAg carriers have normal neopterin levels [2]. Daito et al [17] have reported that neopterin levels and rates of abnormal serum and urinary values in asymptomatic HBsAg carriers did not differ from those of controls, but in the acute hepatitis, neopterin levels were significantly higher than those normal subjects. Serum neopterin levels in patients with B viral chronic liver disease were significantly higher than those in asymptomatic HBsAg. Kiliç et al [18] have reported that neopterin levels were significantly increased in HBV carriers without viral replication as compared with that of controls. In another study, the levels of serum neopterin in patients with hepatic cirrhosis have been found significantly higher than the control groups. In cirrhotic patients neopterin concentrations were not affected by the etiology of liver disease [19]. In HCV positive patients, serum neopterin levels were measured in several studies [20-22].

Grüngreiff et al [21] have found that elevated concentrations of the neopterin in patients with chronic hepatitis C infection before therapy (10.79 ± 5.51 nmol/L) compared to healthy controls (1.25 ± 0.26 nmol/L) but, the difference was found negligible between responders and non-responders during in the IFN therapy. The end of therapy mean concentration was the lowest in partial responders, and highest in non-responders. Neopterin levels in healthy control in their study were lower than our study. We can speculate that these differences may be due to using different kits. Leonardi et al [22] found that serum neopterin was higher in HCVAb positive than in HCVAb negative patients. In another study, neopterin increased from 1 to 2 weeks after the start of IFN-γ in HCV-RNA positive patients [20].

In this study, there was no correlation between neopterin concentrations and HBV DNA levels in the replicative group. It can be concluded that the neopterin level could be related to the presence of the replication and not to the quantity of the viral load. As the neopterin is produced as a result of macrophage activation, neopterin level found in this study to be higher in replicative group as compared to nonreplicative and the control groups indicates that cellular immune response continues in replicative group. However, in this study, absence of increase in neopterin serum levels in all replicative individuals, and the absence of relation between the increase in neopterin levels and the viral load, can derive from different immune response of the host. Even though several other factors may cause neopterin increase, in this study the rise of neopterin levels until to 70% of the replicative group seem very significant.

In conclusion, in the Hepatitis B infected carriers, elevated neopterin levels may be an indicator of the presence of replication. Further studies may need to be carried out to establish the relation between the increase in serum neopterin levels and virus replication in Hepatitis B carriers.

## Competing interests

The author(s) declare that they have no competing interests.

## Authors' contributions

IK participated in its design and coordination of the study, and co-drafted the manuscript. MD participated in the analysis and interpretation of the data and co-drafted the manuscript NC participated in the analysis and interpretation of the data MY participated in interpretation of the data. SD participated in interpretation of the data and co-drafted the manuscript.

## Pre-publication history

The pre-publication history for this paper can be accessed here:


